# Anomalous Origin of the Right Coronary Artery from the Midportion of the Left Anterior Descending Artery: A Rare Coronary Anomaly

**Published:** 2016-07-06

**Authors:** Arash Gholoobi

**Affiliations:** Atherosclerosis Prevention Research Center, Mashhad University of Medical Sciences, Mashhad, Iran.

**Keywords:** *Congenital abnormalities*, *Coronary vessels*, *Coronary angiography*, *Inferior wall myocardial infarction*

## Abstract

The anomalous origin of the right coronary artery (RCA) as a branch from the left anterior descending artery (LAD) is a very rare variation of the single coronary artery anomaly. The anomalous vessel arises from the proximal or midportion of the LAD and courses anterior to the pulmonary artery trunk in most instances. In this case report, a 61-year-old woman is introduced who underwent coronary angiography following inferoposterior myocardial infarction, in which an anomalous RCA was seen originating from the midportion of the LAD. There was also a separate small artery originating from the right coronary sinus, which was most probably a right atrial branch.

## Introduction

The incidence of coronary artery anomalies detected during coronary angiography is about 1.3% in the largest reported series. Among these coronary anomalies, the single coronary artery is a rare anomaly in which one coronary artery stems from a single coronary ostium from the aortic sinuses. A subtype of this anomaly is the origin of the right coronary artery (RCA) from the proximal portion of the left coronary system (with an incidence of 0.009%). In most instances, the anomalous RCA originates from the left main stem and passes between the aorta and pulmonary artery.^1^ A very rare variant of the single coronary artery anomaly is the origin of the RCA as a branch from the left anterior descending artery (LAD).^2^^-^^9^

We herein introduce a patient with a single left coronary artery and associated anomalous origin of the RCA from the midportion of the LAD.

## Case Presentation

A 61-year-old woman was referred to our center for urgent coronary angiography due to failed thrombolysis and ongoing chest pain. She had been previously admitted to a hospital in another city far away from our center due to acute inferoposterior myocardial infarction (MI). She had no known atherosclerotic risk factors, but laboratory examination revealed dyslipidemia with high serum triglyceride (230 mg/dl) and low high-density lipoprotein cholesterol (36 mg/dl). On admission, hemodynamics was stable and chest pain had subsided. On electrocardiography, there were low-voltage limb leads with small q waves in the inferior leads and a pattern of incomplete right bundle branch blockage with an R/S ratio greater than 1 in lead V3 in the precordial leads, altogether indicating a recent inferoposterior MI. Echocardiography revealed inferoposterior wall akinesis with a left ventricular ejection fraction of 40%. 

**Figure 1:**
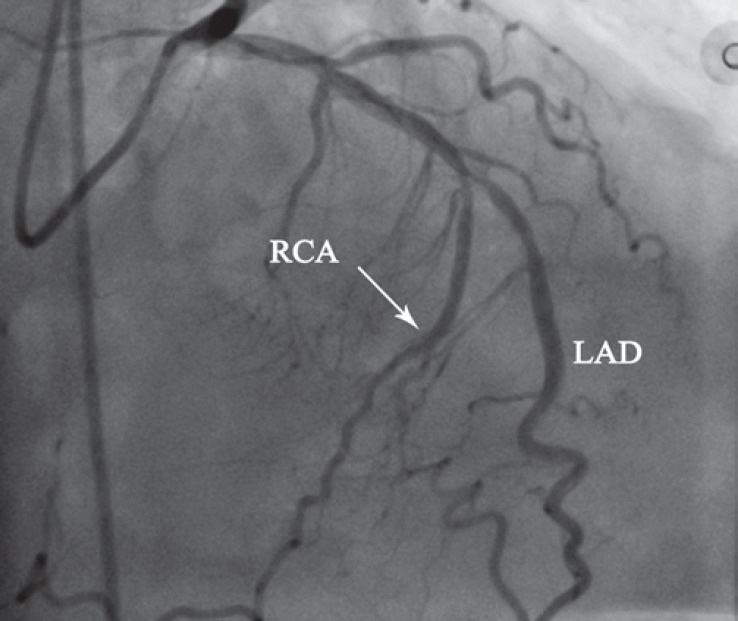
Coronary angiography in the anteroposterior projection with cranial angulation demonstrates severe long stenosis (up to 90%) at the midportion of the left anterior descending artery (LAD). An anomalous vessel is seen originating from the midportion of the LAD (shortly after the second septal perforator within the diseased segment), which follows the course of the right coronary artery (arrow).

RCA, Right coronary artery; LAD, Left anterior descending artery

**Figure 2:**
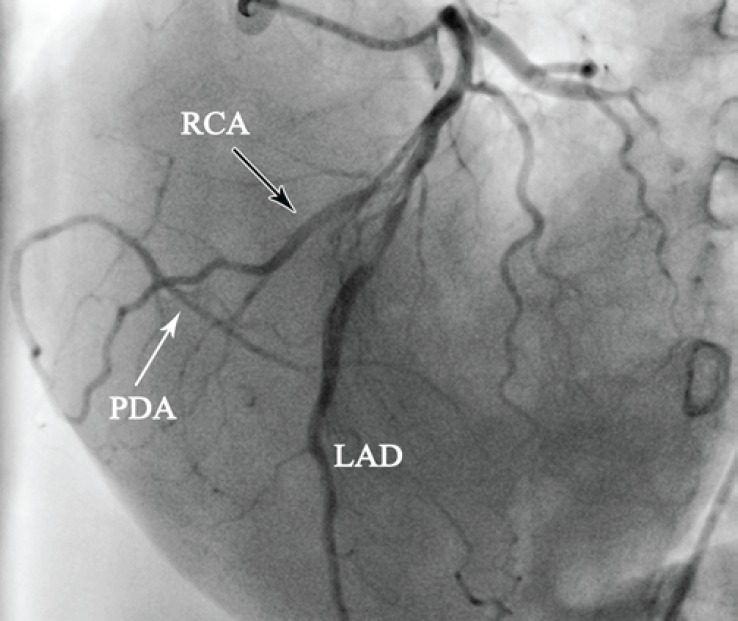
Coronary angiography in the left anterior oblique projection with cranial angulation demonstrates an anomalous vessel originating from the midportion of the left anterior descending artery (black arrow), which travels along the free wall of the right ventricle into the atrioventricular groove and gives rise to a posterior descending artery (white arrow).

RCA, Right coronary artery; PDA, Posterior descending artery; LAD, Left anterior descending artery

**Figure 3:**
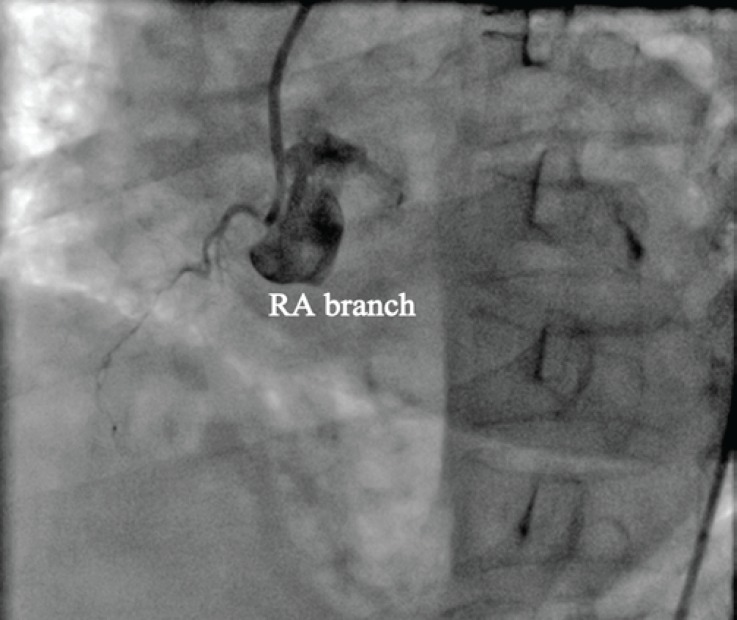
Coronary angiography in the straight left anterior oblique projection demonstrates a small artery originating from the right coronary sinus that most probably is a right atrial branch

**Figure 4:**
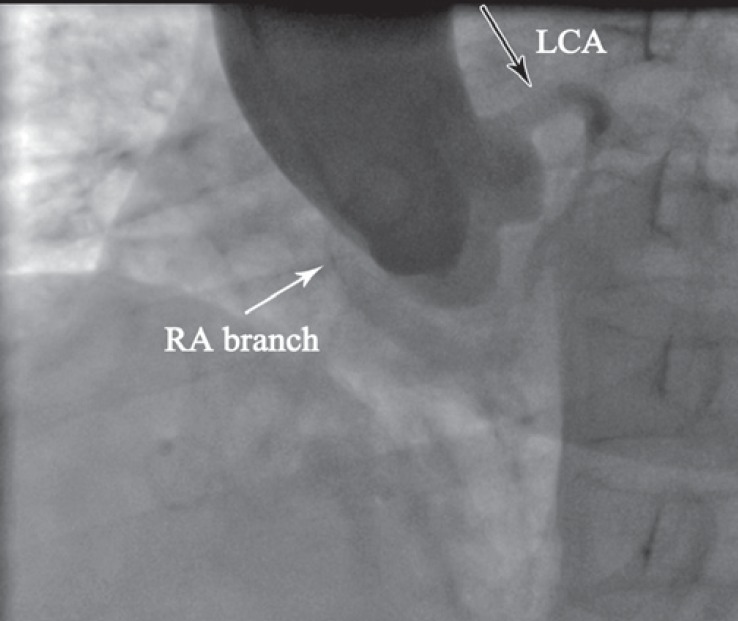
Aortic root injection in straight left anterior oblique projection angiography demonstrates a single major coronary artery originating from the left coronary sinus (black arrow) and a separate small artery originating from the right coronary sinus (white arrow).

The patient subsequently underwent coronary angiography. The left circumflex artery (LCx) was cut off after the first obtuse marginal. The LAD had severe long stenosis (up to 90%) at midportion. An anomalous vessel was seen originating from the midportion of the LAD (shortly after the second septal perforator within the diseased segment), which followed the course of the RCA (Figure 1). In this case, the anomalous RCA traveled along the free wall of the right ventricle into the atrioventricular groove and gave rise to a posterior descending artery (Figure 2). Interestingly, there was also a separate small artery originating from the right coronary sinus that was most probably a right atrial branch (Figure 3). Aortic root injection confirmed the absence of the RCA stump or any other vessel except for the small right atrial branch in the coronary sinuses as well as the ascending aorta (Figure 4). 

**Figure 5:**
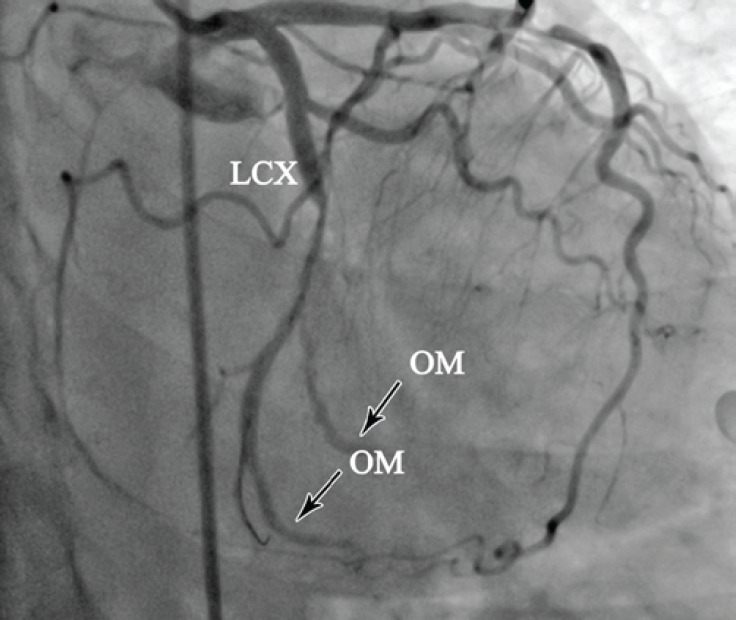
Coronary angiography in the right anterior oblique projection with caudal angulation after predilation, shows 2 large obtuse marginal branches (arrows) at the distal left circumflex artery with poor antegrade flow and heavy atherothrombotic burden.

LCX, Left circumflex artery; OM, Obtuse marginal artery

A decision was made to predilate the LCx lesion using a 1.5 × 15 mm balloon in order to visualize the distal bed. There were 2 more large obtuse marginal branches at distal (Figure 5). Stenting was not performed due to poor antegrade flow and heavy atherothrombotic burden. The patient was put on intravenous Eptifibatide and Heparin and discharged four days later on Aspirin, Clopidogrel, Metoprolol, Captopril, Atorvastatin, and nitrates. Unfortunately, she did not return for follow-up for further percutaneous or surgical revascularization.

RA branch, Right atrial branch

LCA, Left coronary artery; RA branch, Right atrial branch

## Discussion

The anomalous origin of the RCA from the LAD has been reported in the literature as a very rare coronary anomaly. In general, the origin of the anomalous vessel has been from the proximal or midportion of the LAD.^2^^-^^9^ Two courses for this anomalous RCA have been described as retro-aortic or anterior to the pulmonary artery trunk.^2^^-^^4^ All the reported cases have shown the anterior course of the anomalous RCA to the pulmonary artery trunk except for 2 with a retro-aortic course.^5^^, ^^6^ In contrast to the anomalous RCA originating from the left main coronary artery, most cases of which have an inter-arterial course, this malignant course has not been reported until now.^1^^, ^^3^ In all the reported cases, no other associated congenital heart disease has been present in this specific subtype of single coronary artery anomaly except for one patient in association with tetralogy of Fallot.^10^

Based on an extensive search of the literature, the anomalous origin of the RCA from the LAD with a separate small branch arising from the right coronary sinus has not been demonstrated in any previous case reports with the exception of one in which there was also a separate small proximal RCA originating from the right coronary cusp giving rise to a conus, right atrial and right ventricular branch.^7^ In our patient, a separate small artery was found, which was most probably a right atrial branch. 

## Conclusion

The anomalous origin of the RCA from the LAD is a rare coronary anomaly in which the anomalous RCA arises from the proximal or midportion of the LAD and in majority of cases passes anterior to the pulmonary artery before reaching the right atrioventricular groove. It is presumably a benign coronary anomaly and almost always is not associated with other congenital heart diseases.

## Notes:

***This paper should be cited as: ***Gholoobi A. Anomalous Origin of the Right Coronary Artery from the Midportion of the Left Anterior Descending Artery: A Rare Coronary Anomaly. J Teh Univ Heart Ctr 2016;11(3):149-152.
